# Exploring the Molecular Link Between Diabetes and Erectile Dysfunction Through Single-Cell Transcriptome Analysis

**DOI:** 10.3390/genes15121596

**Published:** 2024-12-13

**Authors:** Mahmuda Begum, Mayank Choubey, Munichandra Babu Tirumalasetty, Shahida Arbee, Sibly Sadik, Mohammad Mohabbulla Mohib, Shivani Srivastava, Naofel Minhaz, Riffat Alam, Mohammad Sarif Mohiuddin

**Affiliations:** 1 Department of Internal Medicine, HCA-St. David’s Medical Center, 919 E 32nd St, Austin, TX 78705, USA; mahmuda_b@yahoo.com; 2 Department of Foundations of Medicine, NYU Grossman Long Island School of Medicine, 101 Mineola Blvd, Mineola, NY 11501, USA or choubeymayank48@gmail.com (M.C.); tmunichandrababu@gmail.com (M.B.T.); 3 Institute for Molecular Medicine, Aichi Medical University, 1-Yazako, Karimata, Aichi, Nagakute 480-1103, Japan; shahida.arbee.chobi@gmail.com; 4 National Institute of Preventive and Social Medicine (NIPSOM), Mohakhali, Dhaka 1212, Bangladesh; dr.sibly@gmail.com; 5 Julius Bernstein Institute of Physiology, Medical School, Martin Luther University of Halle-Wittenberg, Magdeburger Straße 6, 06112 Halle, Germany; mohib_nsu007@yahoo.com; 6 Department of Pathology, Yale University, New Haven, CT 06510, USA; shivani.srivastava@yale.edu; 7 PGY1, Family Medicine, University of Alberta, 116 St & 85 Ave, Edmonton, AB T6G 2R3, Canada; minhaz856@gmail.com; 8 Alberta Hospital Edmonton, 17480 Fort Rd NW, Edmonton, AB T5Y 6A8, Canada; alam_riffat@hotmail.com

**Keywords:** diabetes, erectile dysfunction, microvascular complications, diabetic sexual health, men’s sexual health, single-cell analysis, human single-cell transcriptome data

## Abstract

Erectile dysfunction (ED) is a pathophysiological condition in which the patients cannot achieve an erection during sexual activity, and it is often overlooked yet prevalent among diabetic men, globally affecting approximately 35–75% of diabetic individuals. The precise mechanisms through which diabetes contributes to ED remain elusive, but the existing literature suggests the potential involvement of nerve and vascular damage that affects the penile supply. In the present review, we reanalyze the existing human single-cell transcriptomic data from patients having diabetes mellitus-associated ED with normal erections. The analysis validates the expression of genes associated with antioxidative pathways, growth factors, adipokines, angiogenesis, vascular functions, penile erection, sexual function, and inflammation in diverse cell types from healthy individuals and those with ED. Our transcriptomic analysis reveals alterations in the expression of adiponectin receptors in the pathogenesis of ED compared to their counterparts in healthy subjects. This comprehensive review sheds light on the molecular underpinnings of ED in the context of diabetes, providing an in-depth understanding of the biological and cellular alterations involved and paving the way for possible targeted therapeutic discoveries in the field of diabetes-associated male infertility.

## 1. Introduction

**Figure 1:**
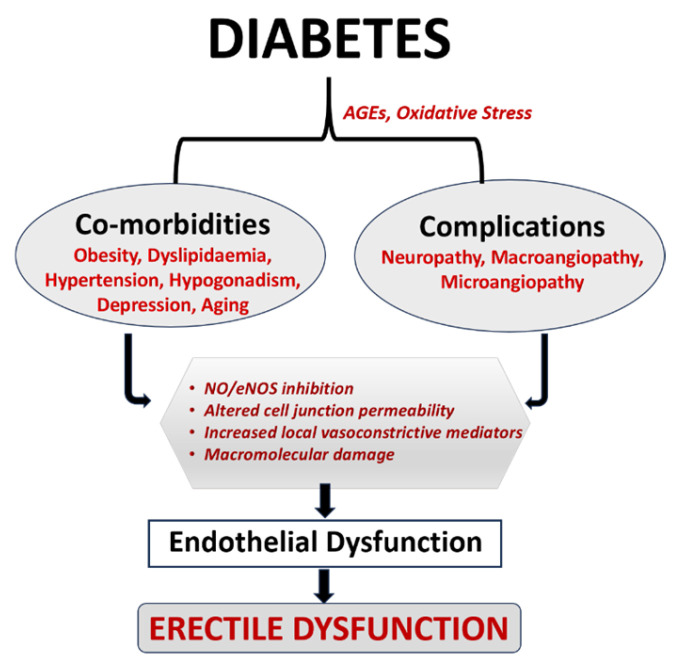
Association of Diabetes and Emergency Department: The hyperglycemic stage of diabetes causes oxidative stress and AGE formation, which further leads to a variety of comorbidities and complications. The aforementioned problems also cause endothelial dysfunction-induced ED through increased local vasoconstrictive mediators, altered cell junction permeability, inhibition of NO/eNOS, and macromolecular damage [18,19].

In the modern world, metabolic disorders are increasing rapidly due to changes in lifestyle, diet, and environmental factors. Diabetes is a common metabolic disorder characterized by insufficient insulin production or peripheral insulin resistance. The number of diabetic patients is increasing day by day, with an estimated 422 million people worldwide [1]. According to the WHO, diabetes is the leading cause of renal failure, cardiovascular events, stroke, visual impairments, and amputation of the lower extremities. In 2019, 2 million deaths occurred due to diabetes and its complications, making diabetes to be one of the deadliest noncommunicable diseases after cardiovascular diseases (CVDs) and cancer [2]. Insulin resistance, autoimmune diseases, hormonal imbalances, pancreatic damage, and genetic mutations are the known etiology of diabetes. The risk factors contributing to diabetes include obesity, a sedentary lifestyle, aging, hypertension, and hyperlipidemia [3]. Diabetes is associated with microvascular complications (such as diabetic retinopathy, diabetic neuropathy, and diabetic kidney diseases) and macrovascular complications (such as coronary artery blockage and lower limb arteritis) (Figure 1) [4]. People with diabetes have a higher risk of CVD including coronary heart disease (CHD), hypertension, elevated low-density lipoprotein-cholesterol (LDL), and obesity [5].

In addition to its well-known complications, diabetes significantly impacts sexual health, particularly contributing to erectile dysfunction in men. Sexual health is essential for the general health and well-being of people, couples, and families, as well as for the social and economic growth of communities and countries. According to the World Health Organization (WHO), sexual health is a state of physical, emotional, mental, and social well-being related to sexuality; it is not simply the absence of disease, dysfunction, or infirmity [6]. Sexual health is more than just the absence of disease and the presence of positive sexual experiences, intimacy, and well-being. It is a fundamental aspect of overall health and a key component of comprehensive healthcare [7]. It involves having a positive and respectful attitude towards sexuality, seeking pleasure and fulfillment during sexual interactions, and abstaining from discrimination and violence. Erectile dysfunction (ED) is an interconnected concept with sexual health that emphasizes the importance of addressing sexual well-being for individuals experiencing difficulties in attaining or maintaining an erection. It is a common disorder that affects millions of men’s sexual health worldwide [8,9]. The importance and significance of managing sexual disorders, particularly ED, cannot be understated due to its tremendous impact on numerous aspects of the individuals’ lives. ED can be defined as the inability to achieve or maintain sufficient rigidity of the penis for sexual intercourse [10]. Several factors are a part of normal erectile function, such as sexual desire or libido for the partner and enough blood circulation from the iliac artery to the corpora cavernosa, which is responsible for erecting and ensuring the penis is rigid enough for adequate penetration, sperm ejaculation, and a good sense of orgasm [11]. According to the International Committee for Sexual Medicine Consultation, the prevalence of ED was 1–10% in men younger than 40 years, 2–9% among men between 40 and 49 years, increased to 20–40% among men between 60 and 69 years, and reached the highest rate in men older than 70 years (50–100%) [12].

Diabetes can significantly affect intimate health for both men and women. Women with diabetes have a higher incidence of sexual disorders than healthy women. A lack of vaginal lubrication, pain during sexual intercourse, and the inability to orgasm are all symptoms of high or low blood glucose levels. Diabetic women also experience a higher rate of depression, which may cause a low sexual drive [13]. According to the statement of the American Diabetes Association, men and women experience low libido as a result of poorly managed diabetes [14]. Multiple studies have demonstrated that erectile dysfunction (ED) occurs more frequently in men with diabetes than in those without the condition [9]. On average, ED occurs 10–15 years earlier in patients with diabetes compared to those without diabetes. The proposed mechanism behind ED in diabetes includes central and autonomic diabetic neuropathy, endothelial dysfunction, and smooth muscle dysfunction [8]. The involvement of neuropathic and angiopathic changes due to high blood glucose contributes to ED [10]. Intimate relationships and sexual satisfaction can be negatively affected by managing a chronic disease such as diabetes, as these emotions can cause stress, anxiety, and sadness. Furthermore, intimate health can also be negatively affected by diabetes physically and psychologically, which can lead to damage to relationships and communication and emotional connection issues in partnerships.

Men with diabetes are much more likely to have ED than men without diabetes [15,16]. A study of more than 1503 participants by Corona et al. observed that in patients with diabetes, 19.4% have mild, 15.4% have mild-to-moderate, 10.4% have moderate, and 21.6% have severe ED [11]. Another meta-analysis by Wang et al. showed that the overall prevalence of ED in diabetic men without diabetic sexual health complications was 37.4%. Another study of 9858 diabetic men reported that 3534 (35%) were suffering from ED [17].

In this review, we will focus on the evidence linking diabetes and ED, the treatment options, research, and advances in diabetes-related ED, as it is very important to emphasize sexual health along with the management of ED that allows people to approach their sexual well-being holistically. Promoting overall sexual well-being not only improves one’s quality of life but also enhances one’s reproductive health, which plays a crucial role in maintaining a stable human population.

## 2. Understanding Diabetes

Diabetes is a chronic health condition characterized by elevated blood sugar levels. Our bodies convert carbohydrates from food into glucose, which serves as our main energy source. Insulin is a hormone made by the pancreatic beta cells that helps glucose to enter cells to be used for energy. In diabetes, the pancreas does not produce any insulin at all or uses it improperly. Glucose then stays in the blood and does not reach your cells.

### 2.1. There Are Three Main Types of Diabetes

Type 1 diabetes occurs when the body’s immune system unintentionally targets and kills the pancreatic cells that produce insulin. As a result, the body produces little or no insulin. This type of diabetes often develops in childhood or adolescence, and individuals with type 1 diabetes must receive lifelong insulin therapy to survive [20].

Type 2 diabetes is the most prevalent form of diabetes, representing the majority of cases. In type 2 diabetes, the pancreatic beta cells fail to produce sufficient insulin, and peripheral tissues become resistant to insulin. Obesity, inadequate physical activity, and a high-carbohydrate diet are the primary causes of type 2 diabetes, which can develop at any age but is most commonly observed in adults. The most effective treatments for type 2 diabetes include lifestyle modifications, oral antihyperglycemic drugs, or insulin therapy [21].

Gestational diabetes (GDM) occurs when blood glucose levels rise excessively during pregnancy. GDM typically manifests between 24 and 28 weeks of gestation. Unlike type 1 diabetes, which results from an insulin deficiency, GDM arises from insulin resistance caused by hormonal changes during pregnancy. Postpartum, the symptoms of gestational diabetes generally resolve. In the United States, GDM is observed in approximately 3 to 8% of pregnant women [22].

Diabetes has both acute and chronic complications. It has a significant impact on overall health, impacts several body systems, and can cause several problems if the glycemic index is poorly maintained. Long-term complications such as neuropathy, nephropathy, retinopathy, cardiovascular diseases, peripheral arterial disease, and diabetic foot ulcers can significantly impair the quality of life and increase the risk of morbidity and mortality in diabetic individuals [23].

Diabetic vasculopathy is characterized by increased blood glucose levels, which diminish the elasticity of blood capillaries, resulting in their constriction and reduced blood flow. This decrease in blood and oxygen supply elevates the risk of hypertension and causes damage to both large and small blood vessels. Hypertension, in turn, is a significant risk factor for cardiovascular disease [24].

**Figure 2:**
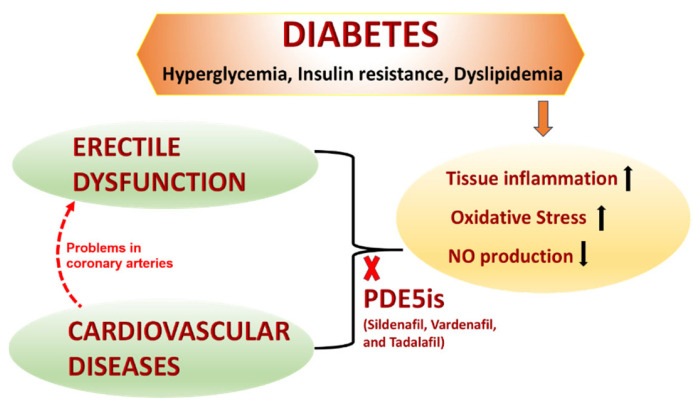
Treatment of diabetes-associated ED. Hyperglycemia, insulin resistance, and dyslipidemia related to diabetes cause oxidative stress, tissue inflammation, and decreased NO production. Commercially available PDE5is, such as Vardenafil, Tadalafil, and Sildenafil, can mitigate this stress-related mechanism, making them an excellent treatment approach for CVD and ED. PDE5is, phosphodiesterase 5 inhibitors; CVD, cardiovascular diseases; ED, erectile dysfunction [31].

Diabetes is an independent risk factor for CVD in both men and women, according to a substantial body of epidemiological and pathological research [25,26,27]. Women with diabetes tend to lose a large part of their natural defenses against getting CVD [28]. Around 65% of people with diabetes have CVD identified as the reason of death [29]. Diabetes is a standalone risk factor for numerous types of CVD (Figure 2), such as heart attacks, strokes, and peripheral artery disease. These disorders are facilitated by high blood sugar levels as well as additional risk factors such as high blood pressure and high cholesterol [30].

Possibly the most significant site of microvascular damage in diabetes is the kidney [32]. Due to their condition and/or additional comorbidities, including hypertension and aging-caused nephron loss, a significant portion of people with diabetes will acquire kidney disease [33]. The prevalence and severity of chronic kidney disease (CKD) can be used to identify those who are more likely to experience unfavorable health outcomes and die young [34].

Nerve complications, specifically diabetic neuropathy, are among the most prevalent complications associated with diabetes. Diabetic neuropathy results from chronic hyperglycemia, which initiates a metabolic cascade involving increased polyol pathway flux, enhanced formation of advanced glycation end products, excessive cytokine release, activation of protein kinase C, and elevated oxidative stress, among other factors. These processes collectively contribute to peripheral nerve damage [35].

The most common and distinctive microvascular consequence of diabetes is diabetic retinopathy, which continues to be the dominant factor in avoidable blindness in persons in their working years [36]. It is identified in one-third of people with diabetes and is associated with a higher risk of fatal systemic vascular consequences such as stroke, coronary heart disease, and heart failure. To reduce the risk of the development and progression of retinopathy, the optimal regulation of blood pressure, blood sugar, and perhaps blood lipids must still be maintained [37].

Patients with diabetes mellitus are more likely to develop infections, which could lead to an increase in morbidity [38]. The hyperglycemic environment that promotes immune dysfunction (such as damage to neutrophil function, depression of the antioxidant system, and humoral immunity), macro- and microangiopathies, neuropathy, a decrease in the antibacterial activity of the urine, gastrointestinal and urinary dysmotility, and a greater number of medical interventions in these patients are what leads to a higher frequency of infections in diabetic patients [39,40]. Mental health issues and the ongoing stress of treating diabetes could exacerbate mental health issues or leave one feeling anxious, depressed, or overwhelmed. According to the Centers for Disease Control and Prevention (CDC), one-third to one-half of diabetics report diabetes-related anxiety over 18 months [41].

### 2.2. Effects of Diabetes on Intimate Health

Diabetes can substantially impact sexual function by affecting both neural and vascular systems. The pathophysiology of erectile dysfunction (ED) in diabetes is multifactorial, encompassing both psychological and organic factors that play a significant role. This condition can affect both men and women, resulting in issues such as erectile dysfunction in men and decreased arousal and lubrication in women [23]. Diabetes also leads to an imbalance of sexual hormones that could affect sexual desire in both men and women. This could result in reduced sexual activity [42]. Women with diabetes experience vaginal dryness due to an impaired blood supply and nerve damage in the vagina, which ultimately causes painful or uncomfortable sexual intercourse and makes intimacy challenging [43]. People with diabetes are more susceptible to infections. Due to high blood sugar, the vaginal pH may be changed, and this allows yeast and bacteria to grow and cause a vaginal infection. Women with a vaginal infection suffer from a foul smell and whitish vaginal discharge that can cause discomfort during sexual intercourse [44]. Diabetes leads to emotional imbalances such as anxiety, depression, and self-esteem problems, which can further impact sexual well-being [45]. Due to diabetic autonomic neuropathy, the sensation in the vagina and penis is reduced, which contributes to the difficulties in achieving arousal and an orgasm [46]. Diabetes also leads to urinary incontinence, which may cause embarrassment and limit sexual activity. Men and women suffering from diabetes also experience infertility. In men, sperm movement reduces while diabetes causes ovulation defects in women [47].

## 3. Erectile Dysfunction

### 3.1. Causes and Symptoms

Erectile dysfunction, also known as ‘impotence,’ refers to the consistent and recurrent inability of a man to achieve and maintain an erection sufficient for satisfactory sexual activity. It is frequently linked to a confluence of emotional, psychological, and physical causes. Physical disorders such as diabetes, cardiovascular disease, high blood pressure, obesity, hormone imbalances, nerve damage, and some drugs can all have an impact on ED [48]. Multiple sclerosis, Parkinson’s disease, and spinal column or pelvic region injuries are examples of neurological conditions that can interfere with erection-inducing nerve signals [49]. Certain medications such as antihypertensive drugs (such as hydrochlorothiazide- Benazepril, Chlorthalidone, etc.), antidepressants, antianxiety drugs, and antiepileptic drugs (such as Fluoxetine, Tranylcypromine, etc.), antihistamines, non-steroidal anti-inflammatory drugs, Parkinson’s disease medications, antiarrhythmics, histamine H2-receptor antagonists, muscle relaxants, prostate cancer medications, and chemotherapy drugs are all implicated in the development of ED [50]. Unhealthy lifestyles such as smoking, inactivity, poor diet, being overweight or obese, metabolic syndromes, and binge drinking are all modifiable risk factors for ED [51,52,53]. Stress and anxiety are mental health issues that can disrupt the brain’s communication with the body, leading to physical responses. Specifically, stress and anxiety can hinder the brain’s ability to signal the penis to increase blood flow, thereby preventing an erection [54].

### 3.2. Symptoms of ED

ED may be a serious indicator that a man’s vascular system is becoming blocked, which is a sign of cardiovascular disease. According to several studies, men with ED are significantly more likely to have a heart attack, stroke, or leg circulation problems. The most common symptoms of ED are a difficulty in achieving or maintaining a firm enough erection for sexual activity. Due to stress and increased sensitivity, ED can occasionally cause a premature ejaculation. A reduced sexual desire is another symptom of ED [55].

## 4. The Interplay of Diabetes and Erectile Dysfunction

The pathophysiology of ED (Figure 1) in diabetes is multifaceted and is influenced by both psychological and organic variables (which are key contributors to ED), as well as psychological and relational problems, which frequently overlap. Visceral obesity, neuropathy, insulin resistance, and hypogonadism are examples of the postulated mechanisms of ED in diabetic individuals [56].

### 4.1. Vascular Damage

**Figure 3:**
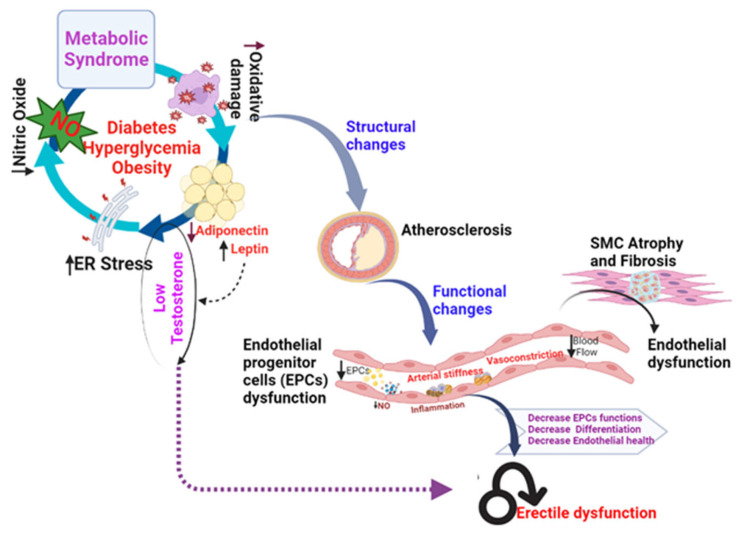
Factors contributing to erectile dysfunction (ED). Endothelial dysfunction leading to ED is attributed to factors such as obesity, stress of the endoplasmic reticulum (ER), and oxidative stress.

Diabetic vasculopathy encompasses macroangiopathy, microangiopathy, and endothelial dysfunction. Microvascular damage in diabetes has the potential to damage smaller blood vessels, including in the penis. Blood flow to the erectile tissues is reduced as a result of this microvascular injury, making it difficult to obtain and maintain an erection [57]. Atherosclerosis, a disorder in which cholesterol and other chemicals build up in the walls of the arteries, narrowing them and limiting blood flow, can be caused by high blood sugar levels. This decreased blood supply to the penile arteries could make it more difficult to achieve and keep an erection [58,59]. Endothelial dysfunction is the term used to describe a variety of clinical disorders, such as altered anticoagulation and anti-inflammatory actions, poor control of vascular development, and dysregulated vascular remodeling. When endothelium-dependent smooth muscle relaxation decreases as a result of decreased or increased nitric oxide (NO) bioactivity in the vasculature, this condition is known as endothelial dysfunction (Figure 3) [60,61,62,63].

### 4.2. Neural Damage

The exact mechanisms underlying diabetic peripheral neuropathy (DPN) remain unclear. However, in both type 1 and type 2 diabetes, hyperglycemia is widely recognized as the primary cause of DPN. The recent literature identifies several potential pathways contributing to the development of DPN, including the involvement of reactive oxygen species (ROS) [64], damage to the microvasculature, a reduction in neurotrophic factors, decreased blood flow to the nerves, decreased neuron integrity, decreased nerve conduction speed, and nerve energy failure, which are all caused by the excessive accumulation of intracellular glucose [65,66]. A group of researchers from Johns Hopkins University School of Medicine conducted a study titled “Association of Peripheral Neuropathy with Erectile Dysfunction in US Men”. The study observed a significant association between peripheral neuropathy and erectile dysfunction [67]. S. Furukawa et al. conducted a study in Dogo with Japanese patients under 65 with type 2 diabetes mellitus. The prevalence of diabetic neuropathy and severe ED in these patients were 47.0% and 39.0%, respectively [68].

### 4.3. Diabetic Autonomic Neuropathy

Diabetes mellitus patients frequently develop diabetic autonomic neuropathy (DAN), a kind of neuropathy marked by dysfunction brought on by damage to the peripheral autonomic nerves. The cardiovascular, gastrointestinal, genitourinary, sudomotor and vasomotor, and neuroendocrine systems are only a few of the many organ systems that can be affected by a wide range of symptoms [46]. The autonomic nerves control involuntary functions, including the relaxation of the smooth muscles in the penis during arousal. Autonomic neuropathy related to diabetes can disrupt this process, making it difficult for the penis to fill with blood and achieve an erection. Quadri et al. conducted a human study and observed that DAN is associated with severe ED [69,70]. A group of researchers from University of Michigan observed men with type 1 diabetes with autonomic neuropathy and also observed ED [71].

### 4.4. Neurotransmitter Imbalance

The blood flow during an erection and detumescence is regulated by peripheral neurotransmitters that are released from the sympathetic nerves (noradrenaline, ATP) and parasympathetic nerves (acetylcholine, nitric oxide, and vasoactive intestinal peptide) that enter the corpora cavernosa, corpus spongiosum, and glans penis [72]. Dopamine, nitric oxide, glutamate, acetylcholine, oxytocin, ACTH, MSH, and pro-VGF are among the neurotransmitters involved in the central regulation of an erection; other neurotransmitters include those that inhibit an erection (such as noradrenaline, enkephalins, GABA, and endocannabinoids), or, as in the case of serotonin, that both facilitate and inhibit erectile function [73]. Type 2 diabetes, characterized by insulin resistance, has been associated with a dopamine imbalance. Kleinridders et al. observed that mice lacking the insulin receptor in the brain displayed a decreased dopamine release in the striatum and involved the disruption of dopaminergic pathways [74].

### 4.5. Chronic Inflammation

In diabetes, due to a continuous elevated blood glucose, chronic inflammation develops. This prolonged hyperglycemia triggers a complicated cascade of immunological reactions involving numerous immune cells, cytokines, and chemokines. Inflammatory mediators such as fibrinogen, C-reactive protein, interleukin (IL)-6, plasminogen activator inhibitor-1, and sialic acid are involved in chronic inflammation in diabetic patients [75]. Although inflammation is a typical reaction to damage or infection, in people with diabetes, this response is dysregulated and contributes to the development of endothelial dysfunction, a sign of vascular dysfunction [76]. Endothelial dysfunction is characterized by a complicated pathophysiology based on the uncoupling of endothelial nitric oxide synthase and the activation of endothelial cells in response to stimulation by numerous inflammatory mediators (molecular patterns, oxidized lipoproteins, and cytokines). These inflammatory reactions ultimately compromise the ability to flow the blood from the penile arteries, which ultimately results in a reduction in successful erections [77]. In addition, chronic inflammatory mediators contribute to the damage of the nerve cells that transport signals from the penis to the brain. As a result, the neural communication required to initiate and maintain an erection can be disrupted. In conclusion, persistent high blood glucose leads to chronic inflammation that creates a hostile environment that compromises both the vascular and neuronal components necessary for normal sexual performance [78].

### 4.6. Oxidative Stress

Oxidative stress is a phenomenon caused by an imbalance between the production and accumulation of reactive oxygen species (ROS) in cells and tissues, leading the biological system to detoxify these reactive products [79]. Although ROS are normally produced as by-products of oxygen metabolism and can play a number of physiological functions (including cell signaling), they are also greatly increased by xenobiotics such as antiblastic drugs and environmental stressors such as UV, ionizing radiation, pollutants, and heavy metals. This imbalance results in cell and tissue damage (oxidative stress). The activation of the hexosamine pathway, nuclear factor-kappa B (NF-κB), p38 mitogen-activated protein kinase (p38 MAPK), c-Jun N-terminal kinase/stress-activated protein kinase (JNK/SAPK), or toll-like receptors (TLRs) results in mitochondrial death under oxidative stress [80]. High glucose levels can cause cells to overproduce ROS, especially in the mitochondria. Oxidative stress leads to damage to DNA, lipids, proteins, and other cellular components. Oxidative stress is crucial in the context of ED because it exacerbates the damage brought on by chronic inflammation [81]. Similar to chronic inflammation, oxidative stress damages endothelial cells directly and alters their ability to produce nitric oxide, resulting in endothelial dysfunction. Furthermore, oxidative stress alters the ratio of ROS to antioxidants in blood vessels, favoring vasoconstriction (the narrowing of blood vessels) and decreasing the relaxation required for optimal blood flow during sexual excitement [82]. Additionally, oxidative stress directly affects the nerves by damaging the myelin covering of the nerve. Oxidative stress can impede the coordination required to achieve an erection by altering the connection between the nerve cells involved in the sexual response and the blood vessels of the penis [83,84].

## 5. Other Risk Factors That Contribute to ED

### 5.1. Obesity

Erectile dysfunction (ED) is usually associated with obesity, which is a major global public health concern. An internal pathologic environment known as common soil may exist between the two disorders. Inflammation, oxidative stress, and the ensuing resistance to insulin and leptin are their primary pathophysiological mechanisms. Furthermore, obesity and comorbid medical disorders, such as ED, are connected in terms of severity [85]. According to De Souza et al., rats with ED caused by high-calorie diets had endothelial damage. Obesity is a distinct risk factor for ED, according to a 14-year prospective research study [86]. Fillo et al. observed that men with abdominal obesity had a higher incidence rate of ED, and the rate increased proportionally to the severity of obesity [87].

### 5.2. Smoking

Cigarette smoking is the leading preventable cause of disease and mortality. Smoking can cause cardiovascular dysfunction and is now recognized as an independent risk factor for the onset of ED, a more serious form of vascular disease. The nitric oxide (NO) pathway, which plays a crucial role in signal transduction, is well understood to be involved in the development of ED [88]. Smoking has been shown to have an impact on both NOS isoforms, the neuronal and endothelial varieties [89]. Smoking harms blood vessels from the inside out, inhibiting elastic dilation even in the presence of potent paracrine signals. As a result of the pathological calcification of elastic fibers caused by smoking, the elastin of the extracellular matrix is changed, resulting in stiffer arteries [90].

### 5.3. Sedentary Lifestyle

The onset ED and diabetes is often influenced by a sedentary lifestyle. Physical inactivity negatively impacts erectile function, while exercise therapies, in both experimental and clinical settings, have been shown to improve sexual responses and overall cardiovascular health [91]. Men who have the symptoms of metabolic syndrome have been reported to have improved erectile performance when eating a Mediterranean-style diet and consuming fewer calories. In addition, in both clinical and experimental studies, the combination of the two interventions has been shown to further improve erectile function [92]. This additional benefit is likely due to a decrease in metabolic disturbances, such as reductions in inflammatory markers and insulin resistance, a decrease in visceral adipose tissue, and improvements in vascular function, including enhanced endothelial function [93].

### 5.4. High Blood Pressure and Cardiovascular Disease

Diabetes is frequently comorbid with cardiovascular disease and high blood pressure (hypertension), which dramatically worsens ED [94]. The endothelium may not be able to provide the required dilation in the vascular bed of the penis in response to sexual arousal, causing a persistent erection impairment, which appears to be the link between the two disorders. On the other hand, there is still room for debate about the true impact of antihypertensive medications on erectile function [95,96]. Some studies have indicated that men with vasculogenic ED who lack the conventional risk factors also have vascular disease, demonstrating the importance of ED as a clinical early cardiovascular risk sign [97].

### 5.5. Psychological Factors

Although psychological factors such as stress, anxiety, and depression are not directly linked to diabetes, they often worsen the effects of erectile dysfunction (ED) [98]. These emotional challenges act as significant barriers, diverting focus away from sexual stimuli and inhibiting arousal. Sex therapy plays a key role in overcoming such obstacles by helping individuals shift attention back to positive sexual cues and reduce the distractions caused by worries or relational conflicts [99]. Cognitive behavioral therapy (CBT) is another effective approach, addressing performance anxiety and negative thought patterns that often accompany ED. This therapy enhances emotional well-being and improves overall relationship satisfaction.

Combining psychological therapies with pharmacological treatments, such as PDE5 inhibitors, has shown promising results in managing ED. While medications address the physical aspects of ED, psychological interventions help address the mental and emotional factors, providing a more holistic approach to treatment. Lifestyle changes, including stress management, exercise, and mindfulness practices, can also play a crucial role in enhancing outcomes. This integrated approach is particularly beneficial for diabetic patients, who often face a combination of physiological and psychological challenges contributing to ED.

## 6. Human Single-Cell Transcriptome Data from NORMAL Erections and ED Patients with DM

Human single-cell transcriptomic data from individuals with DM and those with normal erections offer a thorough and in-depth understanding of the molecular pathways underpinning erectile function and its impairment in diabetic circumstances. The expression of beneficial adipokines, antioxidants, and penile erection genes in single-cell RNAseq datasets related to ED holds promise for unravelling the mechanisms of adiponectin/AdipoRs signaling in this condition, shedding light on its impact on inflammation, angiogenesis, and specific cell populations within the human erectile tissue. Additionally, exploring the expression of other crucial genes associated with ED in reanalyzed single-cell RNAseq datasets comparing normal erectile tissue with ED tissue may aid in understanding the mechanism of diabetes-associated male infertility [100,101]. This knowledge could pave the way for more effective remedies for male reproductive health. Currently, there is a lack of literature on single-cell RNAseq presenting the expression of these important ED-associated genes in various penile cell populations, underscoring the need to identify multiple corresponding pathway genes during ED-linked infertility pathogenesis.

**Figure 4:**
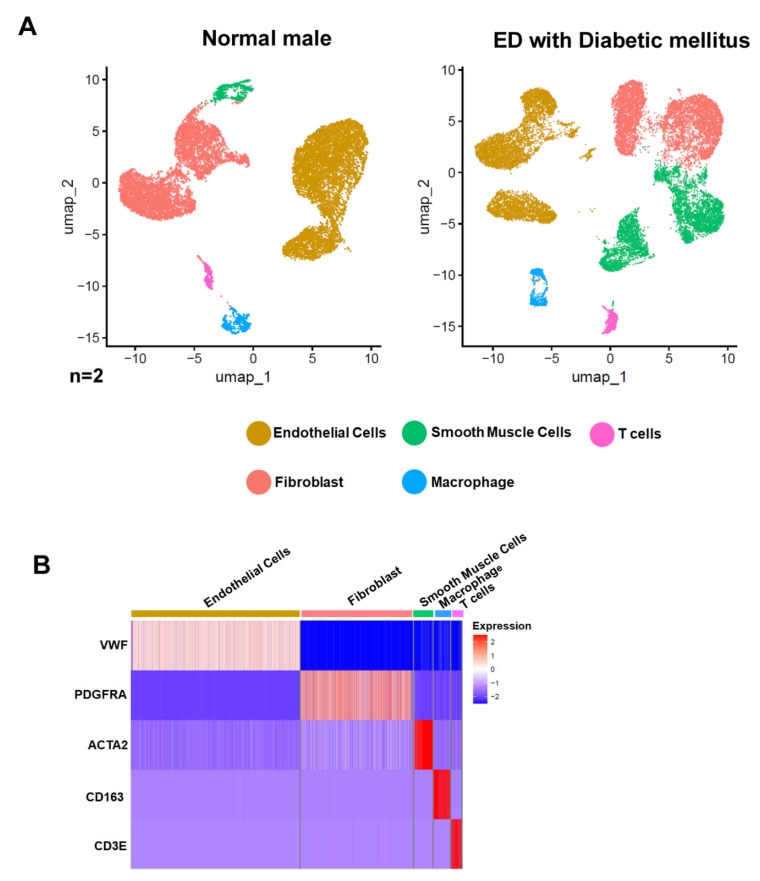
Cellular Composition Analysis in Normal and Erectile Dysfunction (ED) Tissues with Diabetes Mellitus. (**A**) Uniform Manifold Approximation and Projection (UMAP) visualization of single-cell RNA sequencing data from human corpus cavernosum penile tissues comparing a normal male (left) and an ED patient with diabetes mellitus (right). Each dot represents a single cell, color-coded by cell type: endothelial cells (yellow), smooth muscle cells (green), T cells (blue), fibroblasts (pink), and macrophages (red). The number of cells analyzed (n) is indicated below each plot. (**B**) Heatmap depicting the expression profile of selected marker genes across the identified cell types from both conditions. Rows represent individual genes and columns represent cell types as indicated at the top. Color intensity reflects the level of gene expression, with blue indicating low expression and red indicating high expression. Marker genes are VWF for endothelial cells, PDGFRA for fibroblasts, ACTA2 for smooth muscle cells, CD163 for macrophages, and CD3E for T cells. Data were analyzed using the software Seurat v4.1.1 implemented in R v4.2.1.

In our review article, we employed a single-cell analysis to explore the differences between normal erectile human corpus cavernosum tissue and tissues from ED patients obtained from the GEO database (https://www.ncbi.nlm.nih.gov/geo, accessed on 1 February 2024) under accession number GSE206528. Utilizing the Seurat package v4.1.1 [102], we reanalyzed the cellular heterogeneity and landscape utilizing UMAP plots. Additionally, we identified distinct cell types using the established cellular marker genes (Figure 4A,B), previously reported by Zhao et al. [103], revealing various cell types within the corpus cavernosum tissue, including fibroblasts, smooth muscle cells, endothelial cells, macrophages, and T cells.

**Figure 5:**
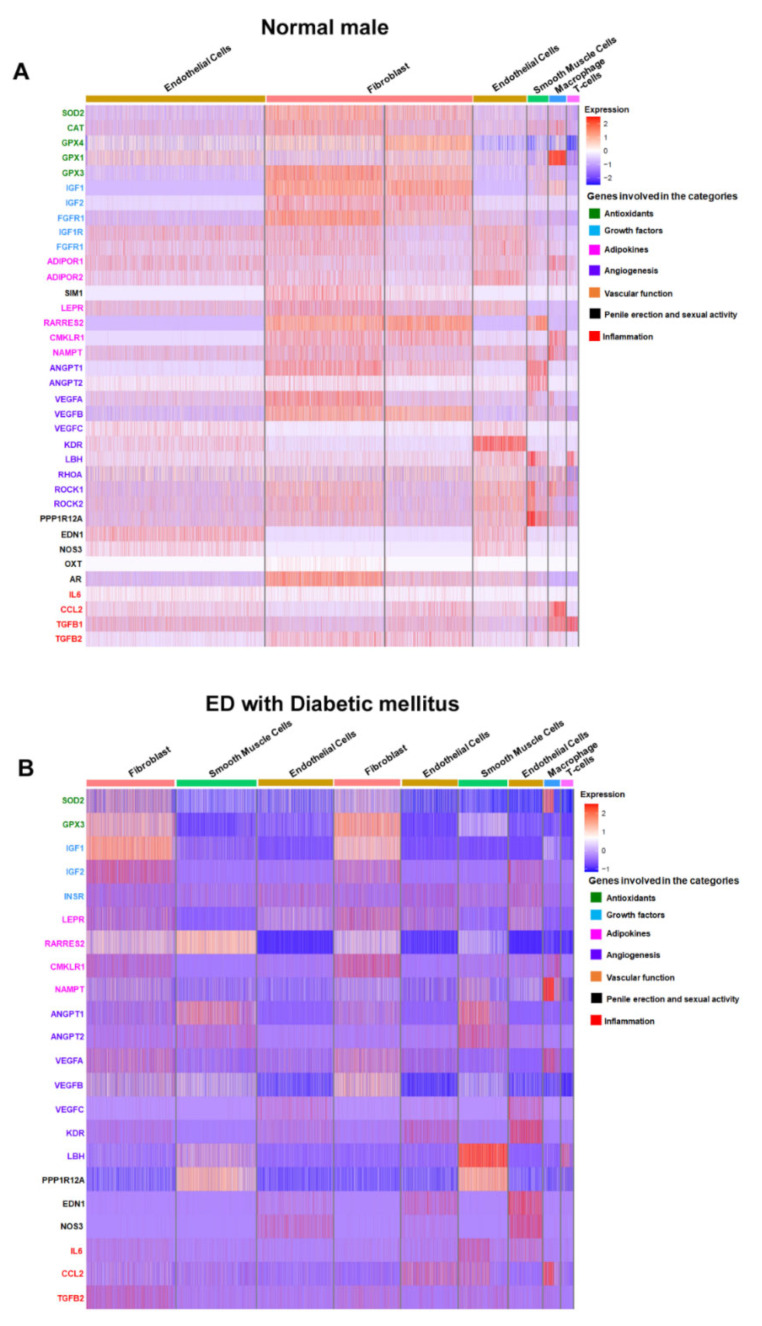
Differential Gene Expression in Penile Tissue of Normal and ED-Affected Males with Diabetes Mellitus. (**A**) Heatmap of gene expression profiles in human corpus cavernosum penile tissue from a normal male. The rows represent individual genes involved in various biological processes such as antioxidants, growth factors, adipokines, angiogenesis, vascular function, penile erection and sexual activity, and inflammation, as color-coded on the right. Columns represent cell types: endothelial cells and fibroblasts. Expression levels are indicated by the color gradient, with red representing high expression, white representing neutral, and blue indicating low expression. (**B**) Comparative heatmap of gene expression profiles in human corpus cavernosum penile tissue from an ED patient with diabetes mellitus. Layout and color-coding are similar to (**A**), showing the same genes and biological process categories across different cell types: endothelial cells, smooth muscle cells, fibroblasts, and macrophages/immune cells.

To elucidate the changes in cellular gene expression during the pathogenesis of ED, we generated a feature heatmap (Figure 5A,B). Our review identified a reduced expression of several genes implicated in angiogenesis, vascular endothelial function, and smooth muscle cell function (e.g., VEGFA, VEGFB, VEGFC, and KDR) in diabetes-associated ED compared to normal subjects. Moreover, we assessed the expression of antioxidative enzymes (SOD, CAT, and GPX) and vascular growth factors along with their corresponding receptors (IGF1/2, FGF1/2, IGF1R, and FGFR1), which exhibited uniformly reduced expression levels. Given the association between ED and decreased serum adiponectin levels [104], our single-cell RNAseq results also revealed a significantly lower expression of beneficial adipokine receptors, including ADIPOR1/R2. Additionally, our analysis highlighted the enhanced expression of certain inflammatory markers (e.g., CCL2 and TGFB1/2) during ED with diabetes mellitus, suggesting a pivotal role of local inflammatory responses in the early pathophysiology of diabetes-associated ED.

## 7. Strategies for Managing and Preventing Erectile Dysfunction in Diabetic Individuals

### 7.1. Medications and Treatments for ED

For erectile dysfunction (ED), there are many treatment options (Table 1) ranging from minimally invasive techniques to more invasive treatments.

#### 7.1.1. Oral Medications (Phosphodiesterase Type 5 Inhibitors orPDE5 Inhibitors)

The four oral PDE5 inhibitors that are commercially accessible in the United States are tadalafil (Cialis, Eli Lilly), sildenafil (Viagra, Pfizer), vardenafil (Levitra and Staxyn, Bayer/GlaxoSmithKline), and avanafil (Stendra, Vivus), a drug that was just recently licensed. Although they can cause adverse effects such as headaches, flushing, nasal congestion, and digestive problems, they are mostly safe and effective [105].

#### 7.1.2. Penile Injection

Platelet-rich plasma (PRP) injections and stem cell therapy (SCT) are two suggested restorative therapies for these problems. SCT involves collecting mesenchymal stem cells or stromal vascular fractions from various tissue sources and injecting them into the target area. Platelet-rich plasma (PRP) is created autologously from a patient’s plasma and then administered by injection into the penile tissue. According to basic science studies, these treatments help restore the damaged penile tissue and promote new cellular and vascular growth. Human studies on SCT have shown promising results [35], and PRP for PD and ED have produced encouraging results with minor negative effects [106].

#### 7.1.3. Vacuum Erection Devices (VEDs)

Vacuum erection devices (VEDs) are conservative, non-invasive, and non-surgical methods for treating erectile dysfunction (ED). To maintain an erection, a constriction ring is positioned at the base of the penis. Men who choose a non-medication approach or those who have not responded well to previous treatments may find that VEDs are an effective alternative [107].

#### 7.1.4. Intraurethral Suppositories

A suppository placed in the urethra is known as alprostadil. The drug promotes blood flow to the penis by being absorbed through the urethral lining. This technique, which is similar to injections, can be successful but can also result in some discomfort or a burning sensation [108].

#### 7.1.5. Natural Therapies

Numerous natural products are available to consumers that claim to revive erections and sexual vitality. Most naturally occurring substances lack sufficient clinical trials to establish their efficacy, according to an analysis of the empirical data that is currently available [109,110]. However, there is some evidence that arginine, yohimbine, Panax ginseng, Maca, and Ginkgo biloba may be beneficial for erectile dysfunction [95,111].

#### 7.1.6. Surgical Interventions

Inflatable or semi-rigid implants are surgically placed within the penis. They provide the person with control over the erection’s timing and length. When inadequate blood flow to the penis is the main cause of ED, surgical treatments can be performed to enhance the blood flow there. Venous ligation surgery involves cutting the veins that allow blood to exit the penis, which helps to maintain an erection.

### 7.2. Management of ED in Diabetic Patients

Maintaining optimal blood sugar control is the key to reducing the risk of erectile dysfunction in people with diabetes. Maintaining normal blood sugar improves blood flow, protects the health of your blood vessels, and reduces your risk of ED. Losing weight is highly recommended to enhance erectile function, as it can increase testosterone levels, primarily by enhancing testicular function and decreasing the testosterone-to-estradiol conversion through aromatase activity in adipose tissue [112]. Physical inactivity is one of the most crucial reversible risk factors. Through many mechanisms, including the regulation of arterial pressure, the synthesis of nitric oxide, the hormone system, and the metabolism of glucose and lipids, regular exercise has been demonstrated to improve erectile function. Furthermore, studies demonstrate that exercise and common medications for the treatment of impotence work together synergistically [113,114].

## 8. Conclusions

This review underscores the intricate relationship between diabetes and erectile dysfunction (ED), highlighting how diabetes significantly impairs vascular and neurological health and contributes to the development and progression of ED. Using insights from single-cell RNAseq datasets (GSE206528), we identified key pathways, such as the reduced expression of genes involved in angiogenesis, oxidative stress regulation, and adipokine signaling, which play a pivotal role in this condition. These findings emphasize the value of single-cell transcriptomics in unraveling cellular complexities and identifying potential molecular targets for therapeutic intervention.

Future research should focus on the experimental validation of these pathways to confirm their role in ED pathophysiology. Expanding the datasets to include diverse populations will enhance the generalizability of the findings. Furthermore, integrating the single-cell data with clinical phenotypes could lead to more personalized treatment strategies. Exploring a combination of targeted molecular therapies and psychological interventions may offer a comprehensive approach to managing ED in diabetic patients. By translating these molecular insights into actionable clinical applications, significant advancements in the treatment of diabetes-related ED can be achieved.

## Figures and Tables

**Table 1:** Outlining some common treatments for erectile dysfunction (ED).

Treatment Option	Description	Advantages	Disadvantages
Oral Medications (Phosphodiesterase-5 Inhibitors)	e.g., Sildenafil (Viagra), Tadalafil (Cialis)	-Effective for many men	-Side effects (headache, flushing, etc.)
Intracavernosal Injections	e.g., Alprostadil	-Rapid onset of action	-Requires self-injection
Vacuum Erection Devices	Mechanical devices that create a vacuum to draw blood into the penis	-Non-invasive	-May cause bruising or discomfort
Penile Implants	Surgical placement of prosthetic devices	-Provides a more natural erection	-Invasive surgery; potential complications
Testosterone Replacement Therapy	For men with low testosterone levels	-May improve ED in those with low testosterone	-Possible side effects; not suitable for all
Psychotherapy/Counseling	Addressing psychological factors contributing to ED	-Effective for psychogenic ED	-Requires commitment; may not work for all
Lifestyle Changes	Exercise, diet, and lifestyle modifications	-Can improve overall sexual health	-Results may take time; not a quick fix

## Data Availability

Data availability does not apply to this article as no new data were created. Data were retrieved from the GEO database under accession number GSE206528 and analyzed.
